# The cut-off value for HOMA-IR discriminating the insulin resistance based on the SHBG level in women with polycystic ovary syndrome

**DOI:** 10.3389/fmed.2023.1100547

**Published:** 2023-03-10

**Authors:** Aleksandra Biernacka-Bartnik, Piotr Kocełak, Aleksander Jerzy Owczarek, Piotr Stanisław Choręza, Leszek Markuszewski, Paweł Madej, Monika Puzianowska-Kuźnicka, Jerzy Chudek, Magdalena Olszanecka-Glinianowicz

**Affiliations:** ^1^Department of Gynecological Endocrinology, Faculty of Medical Sciences in Katowice, Medical University of Silesia, Katowice, Poland; ^2^Pathophysiology Unit, Department of Pathophysiology, Faculty of Medical Sciences in Katowice, Medical University of Silesia, Katowice, Poland; ^3^Health Promotion and Obesity Management Unit, Department of Pathophysiology, Faculty of Medical Sciences in Katowice, Medical University of Silesia, Katowice, Poland; ^4^Department of Statistics, Faculty of Pharmaceutical Sciences in Sosnowiec, Medical University of Silesia, Katowice, Poland; ^5^Faculty of Medical Sciences and Health Sciences, University of Humanities and Technology in Radom, Radom, Poland; ^6^Department of Human Epigenetics, Mossakowski Medical Research Institute, Polish Academy of Sciences, Warsaw, Poland; ^7^Department of Geriatrics and Gerontology, Medical Center of Postgraduate Education, Warsaw, Poland; ^8^Department of Internal Medicine and Oncological Chemotherapy, Faculty of Medical Sciences in Katowice, Medical University of Silesia, Katowice, Poland

**Keywords:** polycystic ovary syndrome, SHBG, HOMA-IR, cut-off value, receiver-operating characteristic

## Abstract

**Introduction:**

The study aimed to estimate the cut-off value for homeostatic model assessment for insulin resistance (HOMA-IR) discriminating the insulin resistance based on the sex hormones binding globulin (SHBG) level in women with polycystic ovary syndrome (PCOS).

**Materials and methods:**

Data from medical records of 854 Caucasian women diagnosed with PCOS were analyzed. Anthropometric data, fasting plasma glucose, insulin and SHBG levels were measured. HOMA-IR was calculated with a standard formula. The cut-off value was calculated using receiver-operating characteristics.

**Results:**

Circulating SHBG levels below the normal range (26.1 nmol/L) were found in 25.4% of study participants. This subgroup had a significantly higher BMI, fasting glucose and insulin concentrations and HOMA-IR values. Empirical optimal cut-off values for HOMA-IR corresponding to low SHBG levels was ≥2.1 [area under the curve (AUC) 0.73, accuracy 0.65, sensitivity 72.3%, specificity 63.1%, positive predictive value (PPV) 40.0%, negative predictive value (NPV) 87.0%].

**Conclusions:**

Our study suggests that the cut-off point for HOMA-IR discriminating the insulin resistance based on the SHBG level, in young Caucasian women with polycystic ovary syndrome is 2.1, and is consistent with the cut-off value adopted by the European Group for the Study of Insulin Resistance (above 2.0).

## Introduction

Sex hormone binding globulin (SHBG) is a homodimer glycoprotein with a high affinity and specificity for androgens and estrogens (1). It is produced mainly in the liver and its synthesis is regulated mostly by circulating sex hormones and hyperinsulinemia compensating insulin resistance (2–4). Thus, SHBG may be a useful marker of the severity of hepatic insulin resistance and fatty liver that is linked to hepatic insulin resistance. Numerous previously published studies demonstrated that low circulating SHBG levels may serve as a surrogate marker of fatty liver (5–7). It has also been shown that SHBG levels were inversely proportional to the severity of fatty liver, insulin levels and homeostatic model assessment for insulin resistance (HOMA-IR) values (8). Moreover, the expression of *SHBG* mRNA correlated negatively with the accumulation of triglycerides in hepatocytes (9). A meta-analysis confirmed these observations, showing that low SHBG levels correlate with non-alcoholic fatty liver disease (NAFLD) in both women and men (10). One of the consequences of hepatic insulin resistance in NAFLD is increased gluconeogenesis resulting in the impaired fasting glucose level. Concurrently, the lower SHBG level is the predictor of type 2 diabetes (11). During a 5 years follow-up, men with the lowest SHBG levels had a four-fold higher risk of type 2 diabetes (12). This finding was corroborated by a meta-analysis of 13 prospective, observational studies (13). In a large cohort study including 42,034 women, a higher risk of type 2 diabetes was associated with SHBG levels < 50 nmol/L (14). The role of SHBG in type 2 diabetes development is supported by experimental studies performed with the insulin-resistant human trophoblast cells (HTR8-SVneo cell line) characterized by low expression of *SHBG, GLUT-3* and *GLUT-4* (glucose transporters type 3 and 4) as well as high expression of *GLUT-1*. Notably, overexpression of SHBG inhibited levels of *GLUT-1* mRNA and promoted the expression of *GLUT-3* and *GLUT-4*. This finding suggests that SHBG may affect glucose metabolism and induce insulin resistance by regulating the activity of glucose transporters (15). In addition, incubation of macrophages and adipocytes with 20 nM SHBG significantly inhibited the synthesis of proinflammatory cytokines (monocyte chemoattractant protein-1, tumor necrosis factor and interleukin-6) induced by lipopolysaccharide treatment (16).

Polycystic ovary syndrome (PCOS) is defined as multiple endocrine and metabolic disturbances, among which the central position is ovarian dysfunction. Insulin resistance is one of the key factors in the pathogenesis of hormonal and metabolic disturbances observed in women with PCOS. However, it should be noted that insulin resistance is not a part of PCOS diagnosis. A gold standard for the assessment of insulin resistance is the hyperinsulinemic-euglycemic clamp technique. However, this method is very complicated and is not used in daily clinical practice. In clinical studies and daily practice, insulin resistance is assessed on the basis of a mathematical model named HOMA-IR, which probably reflects more hepatic than muscle insulin resistance (17). However, there is a lack of a clearly defined cut-off point for HOMA-IR related to insulin resistance. Among many of the proposed values for the general population, the value of 2.5 and above is most often used (18). Notwithstanding, studies performed in Caucasian and Thai women with PCOS suggested the HOMA-IR cut-off value of at least 2.0 (19, 20). Also, the European Group for the Study of Insulin Resistance uses the same cut-off point (≥2.0) (21).

As mentioned above, compensatory hyperinsulinemia inhibits hepatic SHBG synthesis. Concordantly, we hypothesized that SHBG level may be a useful marker of the severity of hepatic insulin resistance. Contrary to the detectable cut-off point characterizing insulin resistance, the laboratory assays for SHBG have specified reference ranges and its lower limit may be used to establish a corresponding HOMA-IR cut-off point. Therefore, the aim of this study was to estimate the cut-off value for HOMA-IR discriminating the insulin resistance based on the SHBG level in women with PCOS.

## Materials and methods

The retrospective study includes data from the medical records of 859 Caucasian women for the first time diagnosed with PCOS on the basis of the Rotterdam criteria (22), hospitalized at the Department of Gynecological Endocrinology from 2012 to 2019.

The inclusion criteria included age 18–30 years and diagnosis of PCOS. The exclusion criteria were: diagnosis of type 2 diabetes and other endocrinological disturbances, any pharmacological therapy, treatment of obesity in the past and currently and the lack of necessary data in the medical records.

The analyzed data set included: age, body mass, height and routine measurements of fasting glucose, insulin and SHBG levels, all performed in a single hospital laboratory using the same set of methods for all study subjects. Glucose concentration was measured using the colourimetric method (Roche reagents for Cobas e111). Insulin and SHBG levels were determined using the ECLIA method (Roche Diagnostic GmbH, Mannheim, Germany reagents for Cobas E411). Body mass index (BMI) and HOMA-IR values were calculated with standard formulas:


$$\begin{array}{l} HOMA-IR=fasting\;serum\;insulin\;level(uIU/ml)\\ \;\times fasting\;glucose\;level\;(mg/dL)/405 \end{array}$$

As the retrospective analysis of patients' records does not meet the criteria of a medical experiment, the approval of the Bioethical Committee was not required.

### Data analysis

Women with HOMA-IR values above 10 (*N* = 5)—data outliers, related to non-compliance and to the assessment of measured parameters in non-fasting subjects, were excluded from the analysis. The remaining women were divided according to the lower limit of the SHBG concentration laboratory's reference range for women aged 18–50 years (< 26.1 nmol/L) into a subgroup with concentrations above and below this limit [*N* = 637 (74.6%) and *N* = 217 (25.4%), respectively].

### Statistical analysis

Statistical analysis was performed using STATISTICA 13.0 PL (TIBCO Software Inc., Palo Alto, CA, US), StataSE 13.0 (StataCorp LP, TX, US) and R software (23). Statistical significance was set at a *p* value below 0.05. All tests were two-tailed. Imputations were not done for missing data. Nominal and ordinal data were expressed as percentages. Interval data were expressed as median with lower and upper quartiles. The distribution of variables was evaluated by the W Shapiro-Wilk test and the quantile-quantile (Q-Q) plot. In order to compare two groups with SHBG ≥ 26.1 nmol/L and SHBG < 26.1 nmol/L, the t-Student test for independent data or the U Mann-Whitney test was used, according to data distribution. The homogeneity of variances was assessed by the F Fisher-Snedecor test. The nominal and ordinal data were compared with the χ^2^ test. Correlation between SHGB levels and other variables was assessed with the ρ Spearman rank correlation coefficient. Age adjustment was done with the Spearman rank partial correlation coefficient (package *ppcor* in R). In order to find a cut-off point discriminating the insulin resistance based on the SHBG level, parametric and non-parametric receiver-operating characteristic (ROC) curves were calculated with an area under the curve (AUC) and corresponding sensitivity, specificity, positive and negative predictive value as well as with accuracy of classification. In order to find an optimal, empirical cut-off point value for HOMA-IR, the Youden J statistic (index) was used.

## Results

**Figure 1:**
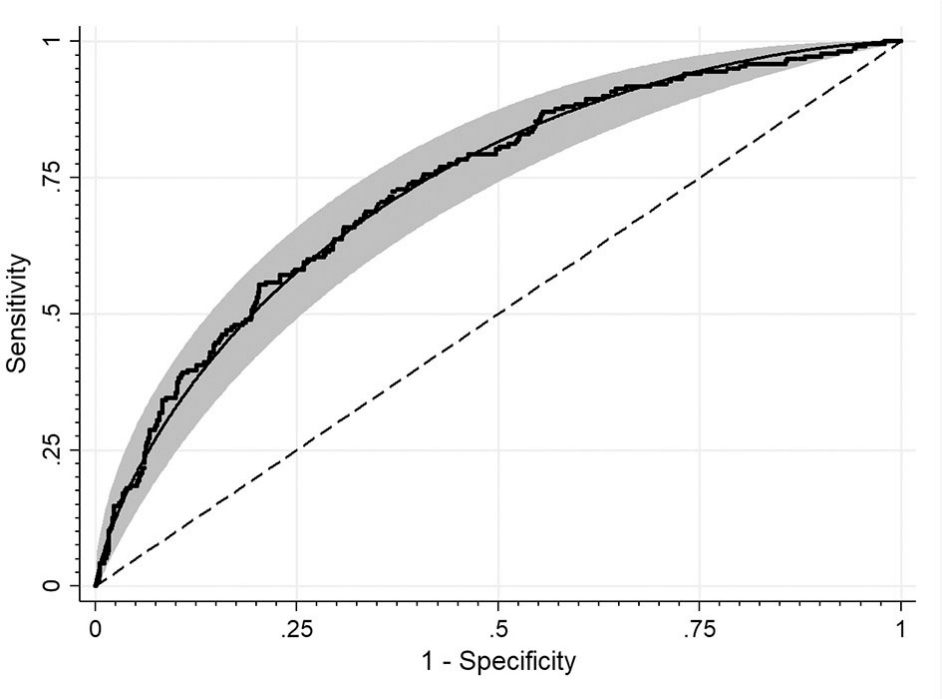
ROC curve for detecting HOMA-IR cut-off values discriminating the insulin resistance based on the SHBG level. The area under the curve: 0.73.

Study groups' characteristics' are listed in Table 1. Circulating SHBG levels below the reference lower limit of 26.1 nmol/L were found in 25.4% of study participants. This subgroup was characterized by a significantly higher BMI, fasting glucose and insulin concentrations as well HOMA-IR values. Obesity and impaired fasting glucose (IGF) were more frequently diagnosed in a subgroup with SHBG below 26.1 nmol/L (59.1% vs. 18.6%; *p* < 0.001 and 17.2% vs. 6.7%; *p* < 0.001, respectively). As expected, the median HOMA-IR value was significantly higher in a subgroup with low SHBG levels (2.8 vs. 1.7; *p* < 0.001). Figure 1 shows the ROC curve of HOMA-IR and SHBG levels below the lower limit of the laboratory reference range (< 26.1 nmol/L). An empirical optimal cut-off, based on the Youden index, for HOMA-IR discriminating the insulin resistance, was ≥2.1 (Table 2). Subjects with HOMA-IR values below the established cut-off had a very low risk of having impaired fasting glucose (OR = 0.035; 95% CI: 0.013–0.097; *p* < 0.001) and decreased SHBG level (OR = 0.19; 95% CI: 0.13–0.27; *p* < 0.001) (Table 3). There was a moderate negative correlation between HOMA-IR values and SHBG levels (crude: ρ = −0.50; *p* < 0.001, age-adjusted: ρ = −0.45; *p* < 0.001), as well as positive with BMI values (crude: ρ = −0.53; *p* < 0.001, age-adjusted: ρ = 0.60; *p* < 0.001).

**Table 1:** Characteristics of the study group and subgroups.

	**All**	**SHBG ≥26.1**	**SHBG < 26.1**
	***N* = 854**	***N* = 637 (74.6%)**	***N* = 217 (25.4%)**
Age (years)^#^	25 (22–29)	25 (22–29)	25 (21–29)
BMI (kg/m^2^) ^#^	26.6 (20.8–31)	22.9 (20.5–27.9)	31.3 (27.1–36.4)^***^
Overweight (*N*; %)	149 (17.4%)	104 (16.3%)	45 (20.9%)
Obesity (*N*; %)	246 (28.8%)	119 (18.6%)	127 (59.1%)^***^
Glucose (mg/dL)^#^	88.0 (83.0–93.0)	88.0 (83.0–92.0)	89.0 (84.0–95.0)^**^
Glucose ≥ 100 (mg/dL) (*N*; %)	80 (9.4%)	43 (6.7%)	37 (17.2%)^***^
Insulin (uIU/ml)^#^	8.9 (6.0–13.2)	7.7 (5.5–11.23)	13.0 (8.9–18.6)^***^
HOMA-IR^#^	1.9 (1.3–3.0)	1.7 (1.1–2.5)	2.5 (2.0–4.3)^***^
HOMA-IR ≥ 2.1 (*N*; %)	386 (45.4%)	235 (36.9%)	157 (72.3%)^***^
SHBG (nmol/L)^#^	39.1 (26.0–59.0)	48.1 (36.0–65.5)	19.3 (15.5–22.4)

^#^Median (lower quartile – upper quartile).

^**^p < 0.01; ^***^p < 0.001; the U Mann-Whitney test for interval data or χ^2^ test for nominal data.

**Table 2:** Sensitivity, specificity, positive predictive value, negative predictive value and accuracy of HOMA-IR ≥2.1 corresponding to low circulating SHBG levels (< 26.1 nmol/L) in PCOS women.

**Parameter**	**Percent (%)**	**95% CI**
Sensitivity	72.3	65.8–78.1
Specificity	63.1	59.2–66.8
Positive predictive value	40.0	35.2–45.1
Negative predictive value	87.0	83.5–89.9

CI, confidence interval.

**Table 3:** Comparison between subjects with lower and higher HOMA-IR using the established cut-off value.

	**HOMA ≥2.1**	**HOMA < 2.1**	***p***
	***N* = 86 (45.2%)**	***N* = 468 (54.8%)**	
Age (years)^*^	25 (22–29)	25 (22–29)	0.85
BMI (kg/m^2^)^*^	30.1 (24.6–35.7)	22.1 (20.1–24.7)	< 0.001
Overweight (*N*; %)	76 (19.7)	73 (15.6)	< 0.001
Obesity (*N*; %)	209 (54.2)	37 (7.9)	
Glucose (mg/dL)^*^	91.0 (86.0–97.0)	86.0 (81.0–90.0)	< 0.001
Glucose ≥ 100 (mg/dL) (*N*; %)	76 (19.7)	4 (0.8)	< 0.001
Insulin (uIU/dL)^*^	13.9 (11.2–18.6)	6.3 (4.7–7.7)	< 0.001
SHBG (nmol/L)^*^	30.8 (20.5–43.9)	48.6 (33.4–67.7)	< 0.001
SHBG < 26.1 (nmol/L) (*N*; %)	154 (39.9)	61 (13.1)	< 0.001

^*^Median (lower quartile–upper quartile).

## Discussion

To the best of our knowledge, this is the first study estimating the cut-off value for HOMA-IR discriminating the insulin resistance based on the SHBG level in women with PCOS.

It is established that HOMA-IR is a better measure of hepatic than muscle insulin resistance. In turn, compensatory hyperinsulinemia inhibits SHBG synthesis in the liver. In our study, 25.4% of women with PCOS had circulating SHBG levels below the adopted lower limit of the laboratory reference range (26.1 nmol/L). This subgroup was characterized by a significantly more frequent occurrence of overweight and obesity diagnosed based on BMI values, according to the Word Health Organization criteria (24), compared to the subgroup with normal SHBG levels. As expected, impaired fasting glucose was also significantly more prevalent in this subgroup, corresponding to a significantly higher median HOMA-IR value (2.9 vs. 1.7). These results, as well as the negative correlations between SHBG levels and HOMA-IR values or insulin levels, once again confirm that low SHBG levels are associated with the occurrence of insulin resistance. These correlations indicate that hyperinsulinemia and insulin resistance explain nearly 50% variability of SHBG concentrations. It is consistent with the results of a previous study analyzing the correlation between SHBG levels and insulin resistance in postmenopausal women (4). Among factors not included in our analysis was hyperandrogenemia exerting a suppressive effect on SHBG secretion, mostly in men (2, 3). However, a meta-analysis of 26 studies including 3,349 menopausal women showed that testosterone but not DHEA administration decreased SHBG levels (25). Thus, hyperandrogenemia potentially may modulate the associations between SHBG levels and hyperinsulinemia also in women with PCOS. However, estradiol/testosterone and estradiol/androstenedione indexes are quite similar in both women with PCOS and obesity and women with PCOS and normal-weight (26). Moreover, 12 months therapy with estrogens, which certainly affects the androgens/estrogens index, did not cause changes in insulin sensitivity in women with PCOS (27). These data suggest that at least the androgens/estrogens ratio has a much less important role than the changes in BMI/fat depot in the modulation of insulin resistance.

In our study, the empirically estimated HOMA-IR cut-off point discriminating the insulin resistance based on the SHBG level below the lower limit of the laboratory reference range (< 26.1 nmol/L) was 2.1. Thus, it is between the previously adopted cut-off points > 2.5 (28), > 2.0 (20, 21) and 1.67 (29). Of note, the HOMA-IR cut-off point determined in our study was characterized by quite high sensitivity but low specificity. Therefore, in many cases the low SHBG level would not allow for the diagnosis of insulin resistance but, on the other hand, the likelihood of false positive results is low. Therefore we do not recommend using SHBG level to diagnose insulin resistance. However, it should be noted that in our subgroup with SHBG levels below 26.1 mmol/L, the prevalence of impaired fasting plasma glucose was about three times more frequent than in a subgroup with SHBG 26.1 mmol/L and above.

Of note, the established HOMA-IR cut-off point in our study of 2.1 is very close to the value of 2.0 in Thai women with PCOS (20). This discrepancy indicates a tightening circle in the search for the optimal HOMA-IR cut-off point for diagnosis of insulin resistance in the population of young women with PCOS. In our study, subjects with HOMA-IR values below the established here cut-off value had a very low risk of impaired fasting glucose. These results are in accordance with a previously published study (17) suggesting that our HOMA-IR cut-off point is a good marker of hepatic insulin resistance. Of note, the cut-off point of 2.1 established in our study is similar to the value determined in 833 Chinese women diagnosed with PCOS and components of metabolic syndrome (30). In addition, the median SHBG concentration in this cohort was 27.9 nmol/L (lower quartile 18.8 nmol/L, upper quartile 45.5 nmol/L) (30), so it was close to the lower limit of the laboratory reference range used in our study.

There are some confounders that should be considered when analyzing HOMA-IR values and corresponding cut-off points discriminating the insulin resistance based on the SHBG level. Borai et al. (31) indicated that studies determining the cut-off points for insulin resistance indicators should refer to the method of insulin assessment, because its concentrations may significantly differ depending on the type of used kit. This may be the effect of several factors, such as variable specificity, different calibration settings, and different formulas used to convert insulin units, as demonstrated by a comparison of 11 insulin determination methods by Manley et al. (32). The same authors observed that the distribution of HOMA-IR values differed even twice, depending on the method of insulin assessment (33). This fact can significantly affect the HOMA-IR cut-off point value estimated in different studies. The results of our and other studies cause reflection or the use of only one parameter in the assessment of insulin resistance with no precisely defined cut-off point, which is associated with a high risk of not recognizing this disturbance. As mentioned above, HOMA-IR calculation is highly variable; therefore, requiring a wider analysis of insulin resistance based on various indicators, perhaps including SHBG. This approach is also recommended by the authors of a study analyzing the advantages and disadvantages of various methods of insulin resistance assessment (33).

Our study has several limitations. The main limitation is its retrospective design. It also lacks hyperinsulinemic-euglycemic clamp, oral glucose tolerance test (OGTT), and HbA1c assessments, as well as body composition and visceral obesity (waist circumference) and fatty liver measures. However, the hyperinsulinemic-euglycemic clamp is still missing the reference values and, therefore, should not be used for the identification of subjects with hepatic insulin resistance. Moreover, both the hyperinsulinemic-euglycemic clamp and OGTT better characterize muscle insulin resistance, while HOMA-IR better assesses hepatic insulin resistance, which was the aim of our study (34). Another limitation is not taking into account hyperandrogenemia as a factor influencing SHBG synthesis. However, it has been previously shown that the contribution of SHBG to the variation in HOMA-IR is not dependent on estrogen and androgens levels in postmenopausal women (35). We hypothesize that this observation may also apply to premenopausal women, as recently published data show the similar predictive significance of SHBG levels for the development of insulin resistance in pre- and postmenopausal women (36).

The strength of our study relies on the large size of the study group and the inclusion of a homogenous cohort of young Caucasian women (between 20 and 30 years of age) with PCOS and a wide range of BMI. Of note, the established cut-off point for HOMA-IR may not be universal for all methods of insulin assessment. We think that the established here cut-off value for HOMA-IR, based on SHBG decline, could be useful for clinicians to identify women with PCOS that may benefit from the implementation of interventions such as an increase in physical activity and changes in eating habits to decrease visceral and liver fat accumulation and prevent the development of type 2 diabetes and cardiovascular disease.

## Conclusions

Our study suggests that the cut-off point for HOMA-IR discriminating the insulin resistance based on the SHBG level in young Caucasian women with PCOS is 2.1 and is consistent with the cut-off value adopted by the European Group for the Study of Insulin Resistance (above 2.0).

## Data availability statement

The raw data supporting the conclusions of this article will be made available by the authors, without undue reservation.

## Ethics statement

Ethical review and approval was not required for the study on human participants in accordance with the local legislation and institutional requirements. Written informed consent from the patients/participants or patients/participants legal guardian/next of kin was not required to participate in this study in accordance with the national legislation and the institutional requirements.

## Author contributions

Concept and study design: AB-B, JC, and MO-G. Data collection: PK and PM. Analysis: AO and PC. Data interpretation and final approval and review: PM, MP-K, JC, and MO-G. Manuscript writing: AB-B and LM. All authors contributed to the article and approved the submitted version.
